# TENS improves CFL injury rat and regulates the intestinal microbiota

**DOI:** 10.1371/journal.pone.0319592

**Published:** 2025-04-03

**Authors:** Min Miao, Tong Ma, Ran Chen, Kuan Geng, Zhiqiang Shen, Yan Sun

**Affiliations:** 1 Pharmaceutical College and Key Laboratory of Pharmacology for Natural Products of Yunnan Province, Kunming Medical University, Kunming, Yunnan, P.R. China; 2 The College of Modern Biomedical Industry, Kunming Medical University, Kunming, Yunnan, P.R. China; 3 Clinical Lab, The Second Affiliated Hospital of Kunming Medical University, Kunming, Yunnan, P.R. China; 4 Department of Radiology, The First People’s Hospital of Honghe State, Mengzi, Yunnan, P.R. China; Huazhong Agriculture University, P.R. CHINA

## Abstract

**Aim:**

This study revealed the mechanism of transcutaneous electrical nerve stimulation (TENS) for improving the calcaneofibular ligament (CFL) injury rats by regulating the intestinal microbiota.

**Methods:**

After 1, 2, and 3 weeks of TENS treatment, the improvement of CFL injury rats model and the expressions of IL-1β/NF-κB/IL-17 signaling pathway were measured. Then the intestinal microbiota was analyzed by 16S rDNA sequencing and its functions related to improve CFL injury rat were analyzed.

**Results:**

TENS could improved the athletic ability of CFL injury rats and reduced the expressions of IL-1β/NF-κB by regulating the IL-17 signaling pathway. By 16S rDNA sequencing analysis, the TENS treatment improved the intestinal dysbacteriosisof CFL injury rats and decrease pathogenic bacteria *Ruminococcus* and *Dubosiella*. The changed intestinal microbiota maybe relative with the ankle injury, whereas the increase in probiotics (*Bacteroides* and *Lactobacillus*) was relative with anti-inflammation.

**Conclusion:**

TENS could down-regulate the expressions of IL-1β/NF-κB to improve CFL injury rat. TENS could change the intestinal microbiota of CFL rats and the changed bacteria whose function related to anti-inflammation could improve CFL rat. The intestinal microbiota could become a potential treatment for CFL injury.

## 1 . Introduction

Ankle sprain is the most common orthopaedic disease among all sports injury, about 85% of ankle sprain is lateral ligament injury [1,2]. When the ankle is subjected to varus or eversion violence, the symptoms of ankle sprain are pain, swelling and movement impairment [3]. CFL injury is characterized by inversion with extreme dorsiflexion and impacted long term function, such as mobility and strength [4]. The injury and fractures of ankle always lead to osteoarthritis [5]. In clinic, the treatment of CFL injury is to ease pain, relieve swell, recover function of ankle [6]. At first, the RICE (Rest, Ice, Compression, Elevation) strategy could relieve the ankle joint symptoms during the first 4 to 7 days [7]. Non-steroidal anti-inflammatory drugs (NSAIDs) could ease pain and inhibit inflammation in the short term, but it has the disadvantage of delaying natural healing [8]. The surgical treatment of CFL injury takes a long time, the incidence of postoperative ankle stiffness is higher, and the complications such as ankle injury may occur [9]. In addition, complementary and alternative medicine (CAM), including ultrasound therapy, laser therapy, short-wave therapy, and invasive electrical stimulation, etc., have a positive effect on pain-relieving and anti-inflammation for CFL injury [10–12].

Transcutaneous electrical nerve stimulation (TENS) is an CAM treatment who can deliver mild stimulation to the human body [12]. In general, the TENS stimulation consists of current intensity, frequency and adjustable time [13]. TENS could ease pain for intra-operative and post-operative pain in patients and effectively avoids the side-effects of opioid analgesics [14]. It exerts analgesic effect and ease pain by activating different levels of afferent nerve fibers and inhibiting the dorsal root reflex activity of inflammatory painful muscles [15]. It was reported that TENS could inhibit inflammation of injury, promote wound healing and decrease the serum inflammatory factors of hip replacement patients [16]. Studies found that TENS could promote fracture healing by improving local blood circulation and cell metabolism by accelerating the division and proliferation of bone-forming cells [17].

Intestinal microbiota and its metabolites regulate homeostasis, such as metabolism, maintain barrier steady-state, inflammation and hematopoiesis, etc. [18]. Studies found that the intestinal microbiota regulated osteoclast through the immune system and local inflammatory of bone marrow [19]. Intestinal microbiota affected the “brain-gut-bone” axis of osteoporosis patients and increased mineral absorption, restored and maintained the intestinal epithelial barrier, reduced oxidative stress [20]. In athletes with a history of lateral ankle sprain, chronic inflammation may cause giving-way, perceived instability, and residual symptoms, associated with the relative abundance of *Bacteroides Fragilis* and *Ruminococcus Gnavus* [21]. The intestinal microbiota could directly or indirectly participate in the inflammatory signaling pathway through nutrients, inflammatory immune response and microbial metabolism [22].

In this study, a CFL injury rat model was duplicated and treated with TENS for 1, 2, 3 weeks. After TENS treatment, the improvement of CFL injury rats model and the expressions of IL-1β/NF-κB/IL-17 signaling pathway were measured. Then the intestinal microbiota of CFL rats was sequenced by 16S rDNA analysis and its functions related to improve CFL injury rat were analyzed. This study concentrated on the functions of TENS who can improve CFL injury and the relationship of CFL injury and the changed intestinal microbiota after TENS treatment.

## 2. Materials and methods

### 2.1. TENS

TENS (AR-T1B1, ZL 2020 1 0460706.1, AnRui co., China) setting range was as follows: the voltage peak value from 0.75–47.20 Vpp; the voltage values from 0.375–23.600 v; the current value 0.367–-23.1280 mA; the electric current density 0.1470–9.2512 A/m^2^; the frequency 25 Hz; the sinusoidal waveform. After 3 weeks of surgery and exercise tolerance test, the surface projection areas of tibial nerve, peroneal nerve, sural nerve, and saphenous nerve were stimulated with TENS, 20 min/day for CFL injury rats.

### 2.2 Animals

All animal experiments were approved by the Animal Study Committee of Kunming Medical University (no. SYXK K20200006) and were conducted according to the requirements of NIH Guidelines for care and use of laboratory animals. A total of 36, three-month-old SD female rats (220 ± 10 g) were maintained in standard conditions with a controlled temperature (21–23°C) and a strict 12 h light/dark cycle. All the rats were fed with standard rat chow and allowed free access to distilled water *ad libitum* at all times during acclimatization and experimental treatment periods.

After 7 days of adaptation, 36 rats underwent the amputations of calcaneofibular ligament of the lateral ankle of the right foot (CFL injury rats). Pre-operatively, all animals were fasted for 24 h. Benzylpenicillin sodium (60,000 IU/kg; Harbin pharmaceutical co., China) were administered for consecutive three days after operations. After 3 weeks of surgery and exercise tolerance test, measure the right hind limb retraction and hoarseness in rats with ankle varus angle over 30° and valgus angle over 15° the rat model of CFL injury was successfully established.

The CFL injury rats were in model group with or without treatment of TENS (model control group without treatment of TENS) and three groups with treatment of TENS. Briefly, low intensity of TENS is 3.16 Vpp, 1.58 v, 1.55 mA; medium intensity of TENS is 6.16 Vpp, 3.08 v, 3.02 mA; high intensity of TENS is 12.90 Vpp, 6.45 v, 6.32 mA (Represented as Low, Medium, High intensity of TENS; L, M, H CFL injury label). Every rat were marked by digital ear-marker.

### 2.3 The intestinal sample collection

After 1, 2, and 3 weeks of treatment with TENS, 24 CFL injury rats (randomly from each group) were euthanized with excessive anesthesia (Intraperitoneal injection with 255 mg/kg 1% sodium pentobarbital; Solarbio Ltd., China). 24 intestinal content samples were collected from the colon bag of CFL injury rats, including CFL_C_1/2/3W_1/2, CFL_L_1/2/3W_1/2, CFL_M_1/2/3W_1/2, CFL_H_1/2/3W_1/2, for 16S rDNA analysis of the intestinal microbiota. All samples were placed in sterile PBS and transported to BGI-Kunming (BGI co., China) and stored at −80°C.

### 2.4 16S rDNA amplicon sequencing

cDNA was extracted from samples, and the V4-V5 region of the 16S rDNA genes was amplified by polymerase chain reaction (PCR) with a primer (341F: ACTCCTACGGGAGGCAGCAG; 806R: GGACTACHVGGGTWTCTAAT). PCR was performed using the following conditions: 3 min denaturation at 94°C; 25 cycles of denaturation at 94°C for 45 s, annealing at 50°C for 60 s, elongation at 72°C for 90 s; and final extension at 72°C for 10 min. The amplicons were purified using AMPure beads (Axygen). Barcoded libraries were generated by emulsion PCR and sequenced in a V5 to V4 reverse direction on a 318 chip using the 400 bp sequencing kit of the Ion Torrent Personal Genome Machine system (PGM) according to the manufacturer's instructions [23]. The output sequences of each sample were no less than 50,000 pairs corresponding to 25,000 clean targets, and informatics methods (strategies: PE101/PE150/PE250/PE300, R language packages: QIIM2, ggplots) were applied.

### 2.5 Inclined plane test

After the duplication of CFL injury model, the hind limbs bearing capacity of CFL injury rat was analyzed by measuring the maximum angle at which CFL injury rats stayed on the inclined plate with rubber pad for 5 seconds. The stay time of rats on the inclined plate required for the rats to stay at a certain weight-bearing capacity of hind limbs, and the rats were observed once before operation, after operation, and on the first, third and seventh day of treatment. Observed and recorded before and after the amputation, on the 1st, 3rd and 7th day of treatment.

### 2.6 *Tarlov* rating

The hind limb motor function of the injured rats was analyzed by measuring the free walking of the injured rats on the horizontal open field and scoring according to the hind limb activity state of the injured rats. The hind limb activity of rats was observed once before, after, and on the 1st, 3rd and 7th day of treatment. Observation and care were performed before and after amputation, day 1, day 3, and day 7 after treatment.

### 2.7 Histopathological evaluation

At weeks 1, 2, and 3 following TENS treatment, two sides of the ankle joint were harvested and stored at −20°C. Next, samples were fixed in 10% paraformaldehyde (Gefan Biotechnology Ltd., China) for 14 d, dehydrated and gradually decalcified. Five-micrometre-thick sections were prepared using a Leica RM2245 microtome (Servio co., China) and were stained with HE, Alizarin red (Servio co., China).

### 2.8 Micro-computed tomography (Micro-CT) analysis

Micro-CT analysis was performed according to recent guidelines56 using a SkyScan 1176 micro-CT imaging system (SkyScan, Bruker Ltd., Belgium) with a spatial resolution of 17.75 mm (X-ray source 70 kV/357 mA, 90 kV/270 mA; exposure time 250 ms/360 ms; magnification × 15; 1.0 mm aluminium/0.1 mm copper filter). Volumetric reconstructions and analysis were performed using the built-in software NRecon 1.6 and CTAn 1.8. For the analysis of bone regeneration, the volume of interest was measured by the average greyscale at the specific bone position (minimum to maximum degree: 0–255).

### 2.9 Quantitative RT-PCR

Using the National Center for Biotechnology Information (NCBI) database designed primers and the cartilage tissue of the ankle joint was collected. For quantitative RT-PCR, cDNA was prepared from 2 mg RNA using a Prime Script RT Reagent Kit (TaKaRa Co., Japan) and analyzed with SYBR Green Master Mix (TaKaRa Co., Japan) in an LC480 Real-time PCR System (Roche Ltd., Germany). Data were quantified using the 2^−ΔΔCt^ relative quantitation method and were normalized to beta-actin expression in each sample. The primer sequences as follows: NF-κB (Rattus) F: TGTGAAGAAGCGAGACCTGG, R: TGCTCCTCTATGGGAACTTGAA; IL-1β (Rattus) F: TTGAGTCTGCACAGTTCCCC, R: GTCCTGGGGAAGGCATTAGG.

### 2.10 Western blot

The ankle joint of CFL injury rat was collected and using BCA (ThermoFisher scientific co., USA) measured the total protein concentration. After the determination of protein concentration, SDS-PAGE electrophoresis 200 V, 30 min, transferred to PVDF membrane, blocking solution for 15min, TBST rinsed 3 times for 5 min each. Then develop color by chemiluminescence using ECL kit (Amersham Biosciences Inc., USA), The luminescent meter is exposed and photographed. Protein band intensity was quantified by densitometric analysis using ImageJ software and normalized to the corresponding bands. The antibody as follows: anti-actin (GB15003, Servicebio Ltd., China), anti-IL-1β (26048-1-AP, Proteintech Ltd., China), anti-NF-κB (10745-1-AP, Proteintech Ltd., China).

### 2.11 Data analysis

The 16S rDNA data analysis was used by the R program packages, including QIIME, Vsearch, Usearch, etc. Statistical significance was determined by one-way analysis of variance (*ANOVA test*) with *Tukey’s post hoc test* for multiple group comparisons (SPSS 27.0, USA), and *P < 0.05* indicated statistical significance.

## 3. Results

### 3.1 TENS improves the athletic ability of CFL injury rats

**Fig 1:**
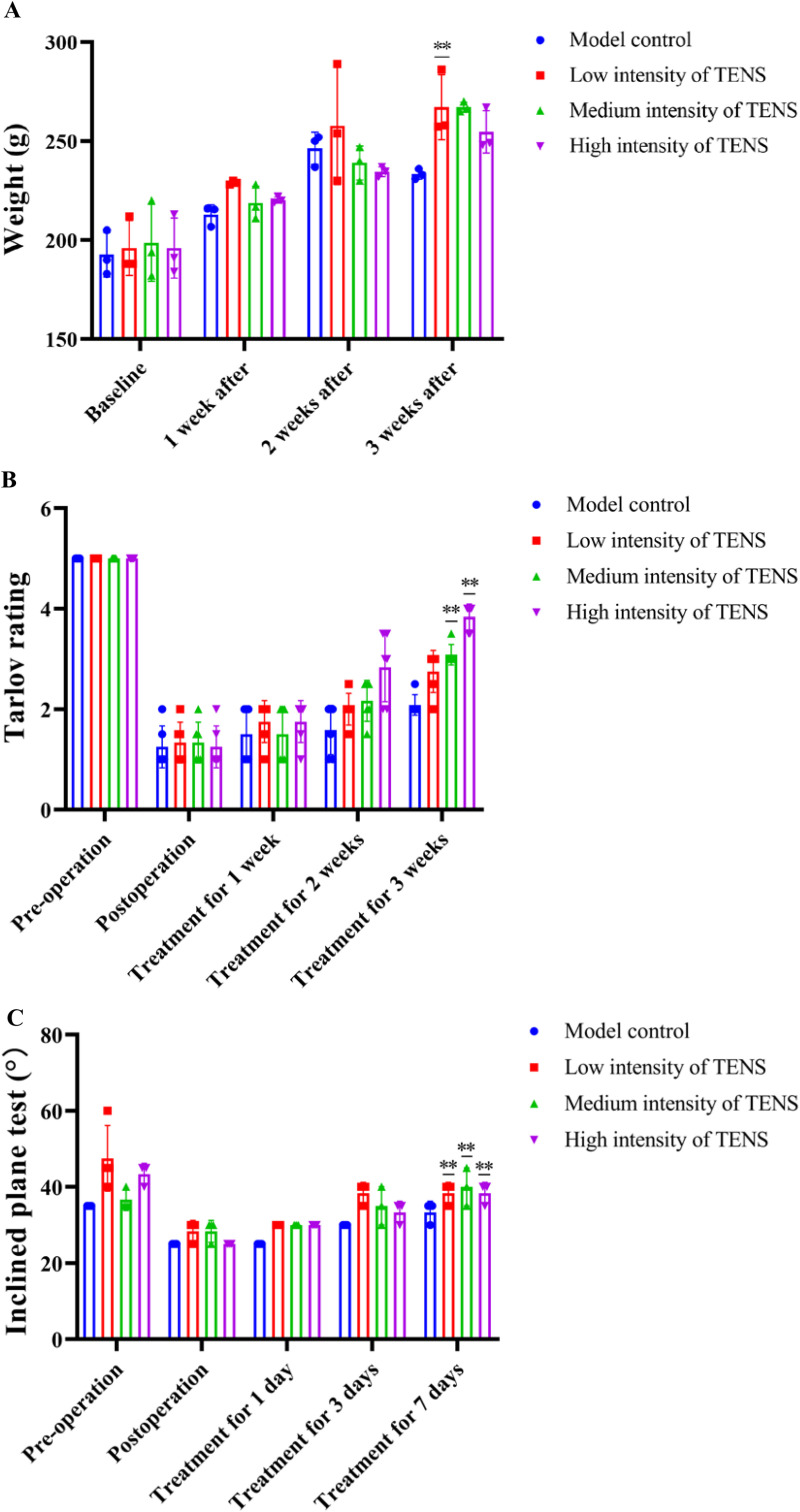
TENS improved the athletic ability of CFL injury rats. A. The weights of CFL injury rats in each group after 1, 2, 3 weeks of treatment with TENS. B. The hind limbs activity state of CFL injury rats in each group before/after ligamentectomy and on the 1st, 3rd and 7th day of treatment with TENS. C. The hind limbs bearing capacity of CFL injury rats in each group before/after ligamentectomy and on the 1st, 3rd and 7th day of treatment with TENS. The values are presented as the mean ± standard deviation (n = 3). Compared to the model control group (CFL injury group),^ * ^*P < 0.05,* ^**^P < 0.01.

After 1 week exercise tolerance test, when the ankle inversion angle of the rat exceeded 30.00° (34.69 ± 0.81°), the eversion angle exceeded 15.00° (18.42 ± 1.64°), the right hind limb retraction and neighing, the CFL injury rat model was successfully duplicated. After 1 week of treatment with TENS, the weight was same as the baseline in each group; After 2 and 3 weeks of treatment with TENS, compared with the model control group, the weight of CFL injury rats increased in the three TENS groups (*P > 0.05*, Fig 1A). After 1 week of treatment with TENS, compared with the model control group, the behavioral capacities increased in the medium and high intensity of TENS groups (*P < 0.05*, Fig 1B). After 1 week of treatment with TENS, compared with post-operation, the load-bearing capacity improved in the three TENS groups (*P < 0.05*, Fig 1C).

### 3.2 TENS inhibits inflammation of CFL injury rats by regulating the IL-1β/NF-κB/IL-17 signaling pathway

**Fig 2:**
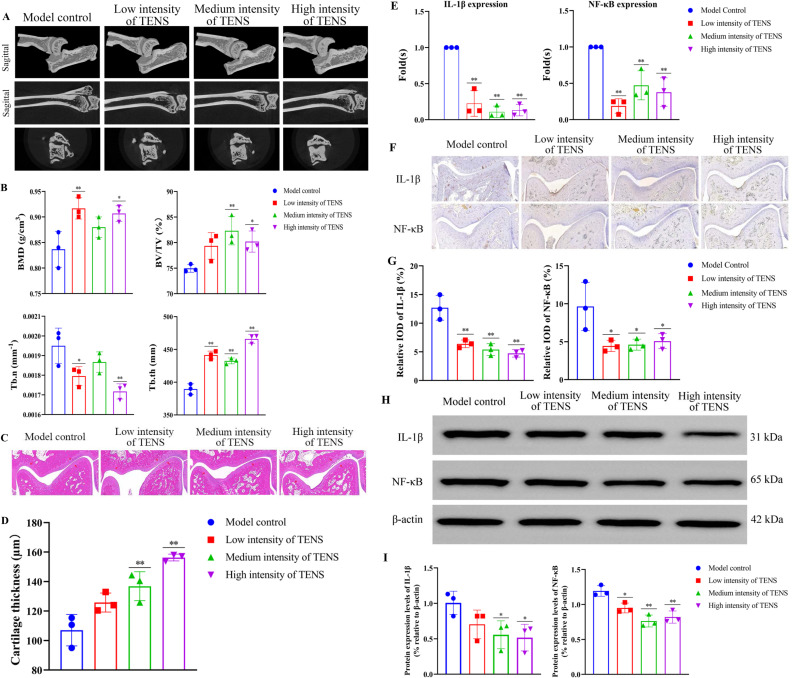
TENS inhibited inflammation of CFL injury rats by regulating the IL-1 β/NF-κB/IL-17 signaling pathway. A. Effect of TENS on CFL injury rats model. B. The analysis of ankle by Micro-CT. The BMD, BV/TV, Tb.n and Tb.th were measured. C&D. The HE staining of distal tibiofibula, calcaneus and talus in each group. E. The relative mRNA expression of IL-1β and NF-κB in each group after 3 weeks of treatment with TENS. F&G. The IHC staining of IL-1β and NF-κB in each group after 3 weeks of treatment with TENS. The green color represented as IL-1β and the red represented as NF-κB. H&I. The expressions of IL-1β and NF-κB in each group which analyzed by WB after 3 weeks of treatment with TENS. The values are presented as the mean±standard deviation (n = 6). Compared to the model control group (CFL injury group), ^* ^*P* < 0.05, ^**^P < 0.01.

After 3 weeks of treatment with TENS, the tissue morphology of distal tibiofibula, calcaneus and talus in each group was completed (Fig 2A); Compared with the model control group, the bone mineral density (BMD), trabecular number (Tb.n) increased in the low and high intensity of TENS groups (*P* < 0.05, Fig 2B); Compared with the model control group, the bone volume fraction (BV/TV) increased in the medium and high intensity of TENS groups (*P < 0.05*); Compared with the model control group, the trabecular thickness (Tb.th) increased in the three TENS groups (*P < 0.05*). After 3 weeks of treatment with TENS, compared with the model control group, the cartilage thickness of calcaneus and talus increased in the medium and high intensity of TENS group (*P* < 0.05, Fig 2C, D). After 3 weeks of treatment with TENS, compared with the model control group, the IL-1β of ankle articular cartilage decreased in the three TENS groups (*P* < 0.05, Fig 2E); the NF-κB of ankle articular cartilage decreased in the low and high intensity of TENS group (*P* < 0.05, Fig 2E); Compared with the model control group, the relative average optical density (AOD) of IL-1β and NF-κB of ankle decreased in the three TENS groups (*P* < 0.05, Fig 2F, G). After 3 weeks of treatment with TENS, compared with the model control group, the expressions of IL-1β and NF-κB decreased in the three TENS group (*P* < 0.05, Fig 2H, I).

### 3.3 16S rDNA operational taxonomic unit (OTU) results of CFL injury rats after TNES treatment

**Fig 3:**
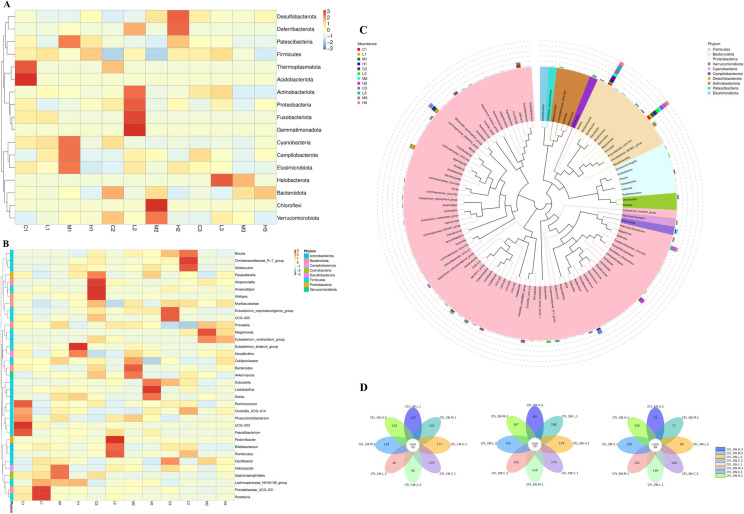
16S rDNA operational taxonomic unit (OTU) results of CFL injury rats after TENS treatment. A. The species abundance clustering heatmap of groups at phylum level. B. Genus level species abundance clustering heatmap. The abscissa in the figure is the sample, and the ordinate is the species annotation information. The cluster tree on the left side of the figure is a species clustering tree; The value corresponding to the heat map is the Z-value obtained after the relative abundance of each row of species is normalized, that is, the Z-value of a sample on a certain category is the difference between the relative abundance of the sample on that classification and the average relative abundance of all samples in the classification divided by the standard deviation of all samples on the classification.). C. The abundance and microbiota of all groups. A horizontal species evolutionary tree, and its color of branche and sector represented as the corresponding phyla. The stacked histograms represented as the abundance distribution of samples at the genus level. D. The OTU distribution of all groups. Each circle represented as a group, the overlapping part of the circle represented as the number of common OTUs in each group.

According to the species annotation and abundance information of all samples at the phylum and genus level, the top 35 phylum and genus in abundance were found. (Fig 3A, B). After 3 weeks of TENS treatment of CFL injury rats, compared with the C3 (Control. 3W. CFL injury) group, the H3 (High intensity. 3W. CFL injury) group increased the species content of *Bacteroidetes*, *Desulfobacterota*, *Proteobacteria* and *Cyanobacteria. Firmicutes*, *Patescibacteria*, *Actinobacteriota*, *Campilobacterota* and *Elusimicrobiota* were significantly reduced at the phylum. After 3 weeks of TENS treatment of CFL injury rats, compared to the C3 (Control. 3W. CFL injury) group, the *Blautia*, *Eubacterium_ruminantium_group*, *UCG_005*, *Dubosiella*, *Oscillibacter* decreased and the *Parasutterella*, *Desulfovibrio* increased in the H3 (High intensity. 3W. CFL injury) group at the genus. After 1 week of TENS treatment of CFL injury rats, compared to the C1 (Control. 1W. CFL injury) group, the intestinal microbiota in the L1 (Low intensity. 1W. CFL injury) group, M1 (Medium intensity. 1W. CFL injury) group and H1 (High intensity. 1W. CFL injury) group were changed (Fig 3B). The microbiota of top three groups were *Firmicutes, Bacteroidota, Preteobacteia* at the phylum level (Fig 3C). The number of core OTUs in the 1W. CFL injury group and 2W.CFL injury group was 25; The number of core OTUs in the 3W.CFL injury groups was 26 (Fig 3D).

### 3.4 Alpha diversity analysis of CFL injury rats after 3w treatment with TNES

**Fig 4:**
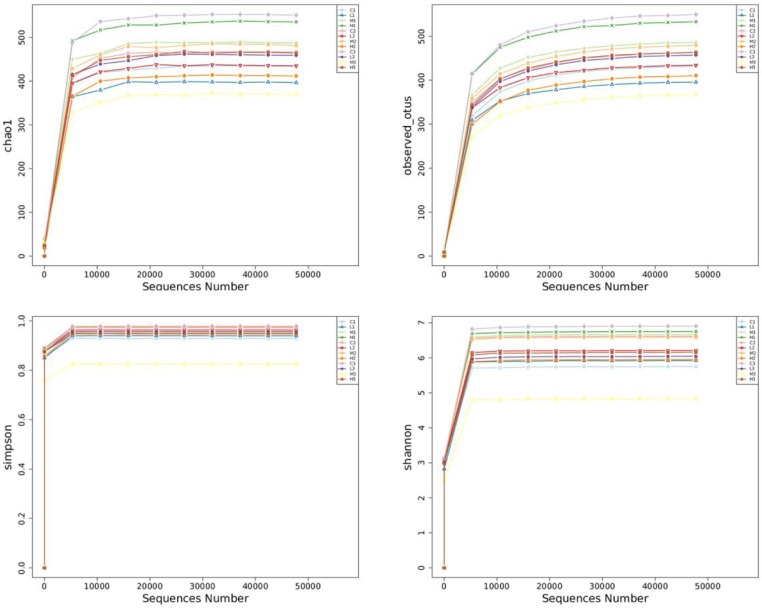
Alpha diversity analysis of CFL injury rats after 3w of TENS. The alpha diversities of CFL injury rats in each group. Community Richness: Chao1 - the Chao1 estimator/Observed_otus - the number of observed species Community Diversity: Simpson - the Simpson index/Shannon - the Shannon index.

The coverage rarefactions of chao1 group, Observed_otus group, shannon and simpson group were greater than 99% (Fig 4). After 3 weeks of TENS treatment, compared with the C3 (Control. 3W. CFL injury) group, the H3 (High intensity. 3W. CFL injury) group decreased the community richness in Chao1 and Observed_otus. The community diversity decreased in Shannon and Simpson index.

### 3.5 Beta diversity analysis of CFL injury rats after 3w treatment with TNES

**Fig 5:**
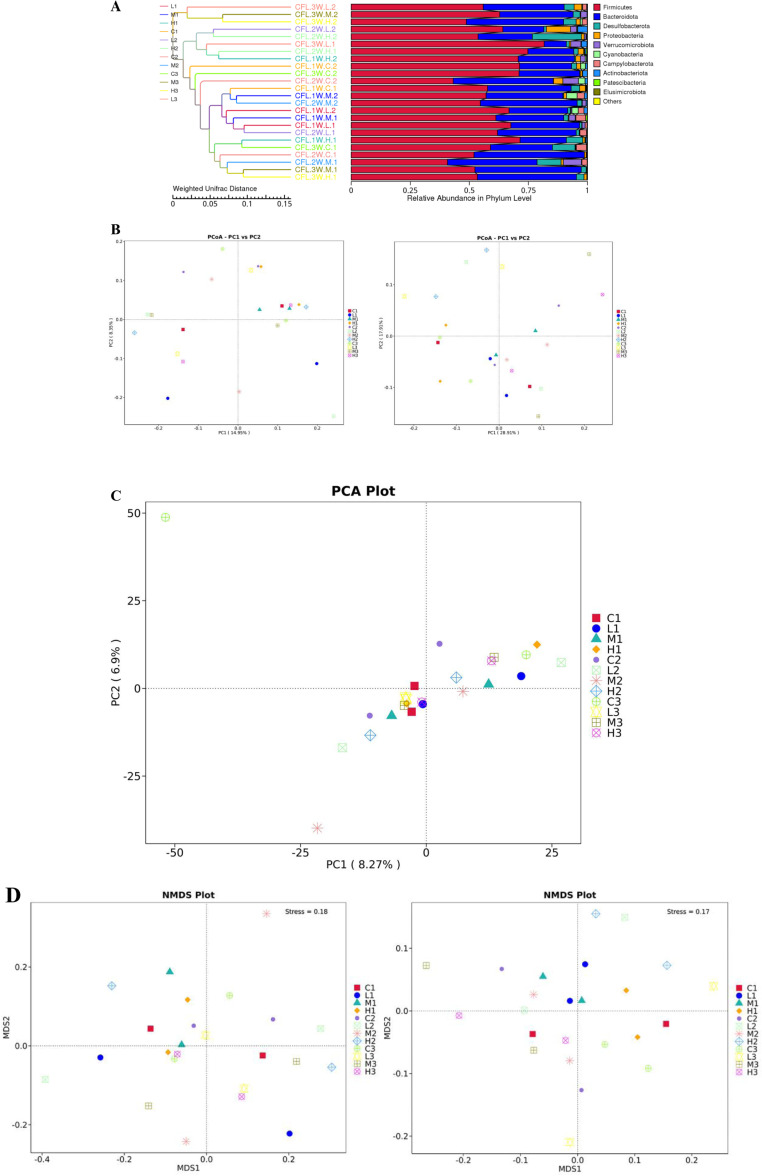
Beta diversity analysis of CFL injury rats after 3weeks treatment with TENS. A. The relative abundance of all samples which analyzed by UPGMA clustering at phylum level. The smaller value of unweighted unifrac and weighted unifrac represented as the smaller difference. B&C&D. The beta-diversities of all samples which analyzed by PCoA, PCA and NMDS. Each point represented as a sample, and samples in the same group were represented as the same color.

The UPGMA (Unweighted Pair-group Method with Arithmetic Mean) results showed that the smallest differences in species diversity between the samples were CFL.1W.L.1 and CFL,2W. L.1, and the largest differences in species diversity were CFL.2W.L.2 and CFL.2W.H.2 (Fig 5A). There were obvious differences between the control samples and the TENS treatment samples and the difference percentages were 28.91% and 17.91% which analyzed by principal coordinate analysis (PCoA), respectively (Fig 5B). The results showed that the data gap between the groups was significant, and the data gap within the group was small. The separation rates of PC1 and PC2 were 8.27% and 6.9% which analyzed by Principal Component Analysis (PCA; Fig 5C). According to the results of NMDS (Non-Metric Multi-Dimensional Scaling) analysis, the separation of plots showed a significant difference between the C3 (Control. 3W. CFL injury) group and the H3 (High intensity. 3W. CFL injury) group (Fig 5D).

### 3.6 Compositions and changes in CFL injury rats at the phylum and genus levels after 3 weeks of TENS treatment

**Fig 6:**
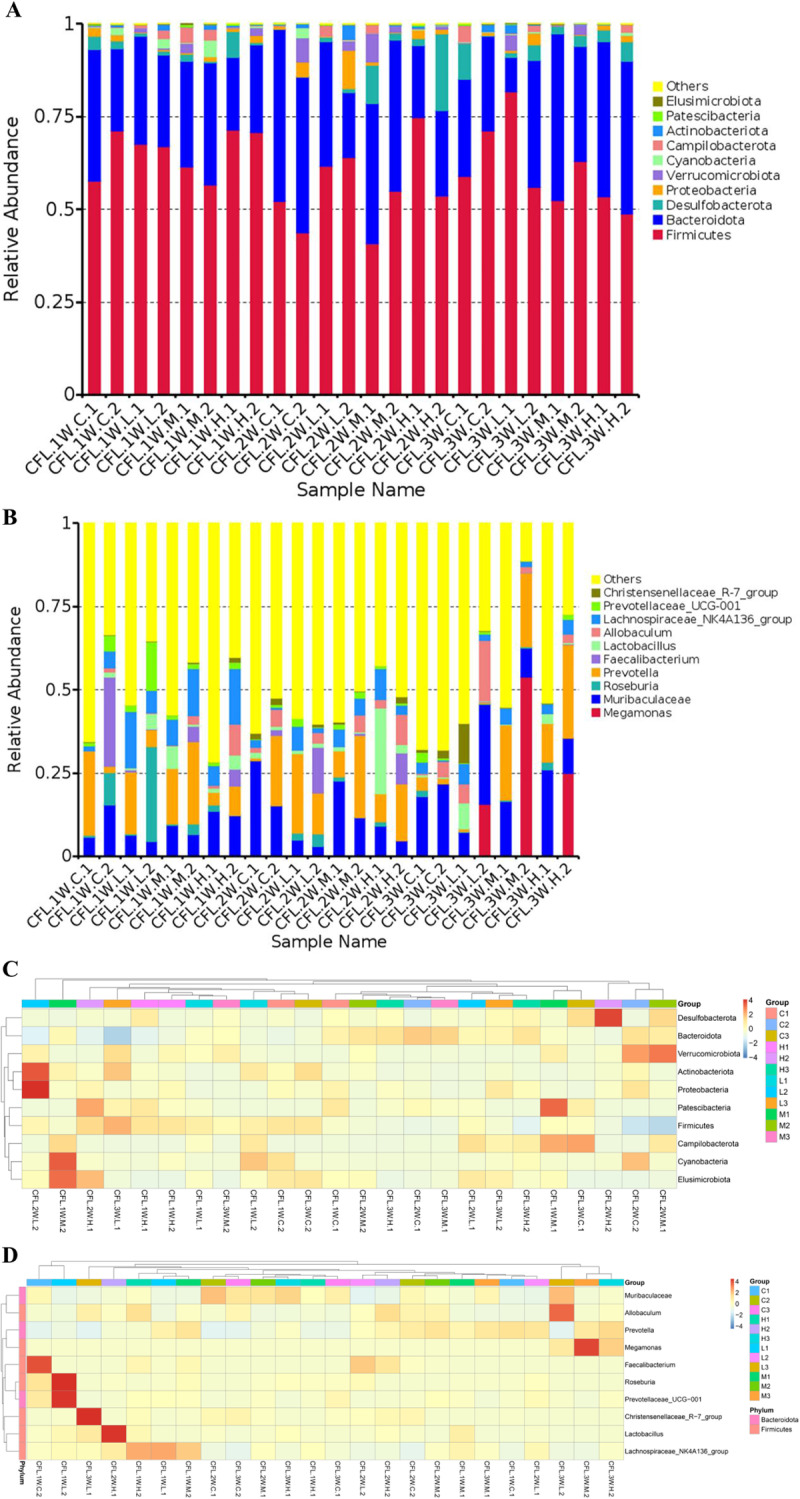
Compositions and changes in CFL injury rats at the phylum and genus levels after 3w treatment of TENS. A&C. The Compositions and changes in CFL injury rats at the phylum level after 3w treatment of TENS. B&D. The Compositions and changes in CFL injury rats at the genus level after 3w treatment of TENS.

After 3 weeks of treatment with TENS, compared with the C3 (Control. 3W. CFL injury), *Patescibacteria*, *Actinobacteriota*, *Elusimcrobiota* decreased in the M/H3 (Medium/High intensity. 3W. CFL injury) groups and *Bacteroidota* increased in the (Medium/High intensity. 3W. CFL injury) groups at the phylum; *Halobacterota* increased in the L/M3 (Low/Medium intensity. 3W. CFL injury) groups and decreased in the H3 (High intensity. 3W. CFL injury) group; *Proteobacteria* increased in the L/H3 (Low/High intensity. 3W. CFL injury) groups and decreased in the M3 (Medium intensity. 3W. CFL injury) group; *Verrucomicrobiota* increased in the L/M3 (Low/Medium intensity. 3W. CFL injury) groups (Fig 6A, C). After 3 weeks of treatment with TENS, there were significantly different microbiota between the C3 (Control. 3W. CFL injury) group and the three intensities of TENS group at the genus. The *Alloprevotella*, *Megamonas*, *Phascolarctobacteriu*, *Lachnospiraceae_NK4A136_group* increased in the L/M/H3 (Low/Medium/High intensity. 3W. CFL injury) groups and *Eubacterium_coprostanoligenes_group*, *UCG-005*, *Dubosiella*, *Oscillibacter* decreased in the L/M/H3 (Low/Medium/High intensity. 3W. CFL injury) groups. Compared with the C3 (Control. 3W. CFL injury) group, *Blautia*, *Christensenellaceae_R-7_group*, *Allobaculum*, *Ruminococcus* decreased in the M/H3 (Medium/High intensity. 3W. CFL injury) groups; *Parasutterella*, *Bacteroides*, *Lactobacillus* increased in the L/H3 (Low/High intensity. 3W. CFL injury) groups and decreased in the M3 (Medium intensity. 3W. CFL injury) group; *Bifidobacterium* increased in the M/H3 (Medium/High intensity. 3W. CFL injury) groups (Fig 6B, D).

### 3.7 KEGG functional analysis

**Fig 7:**
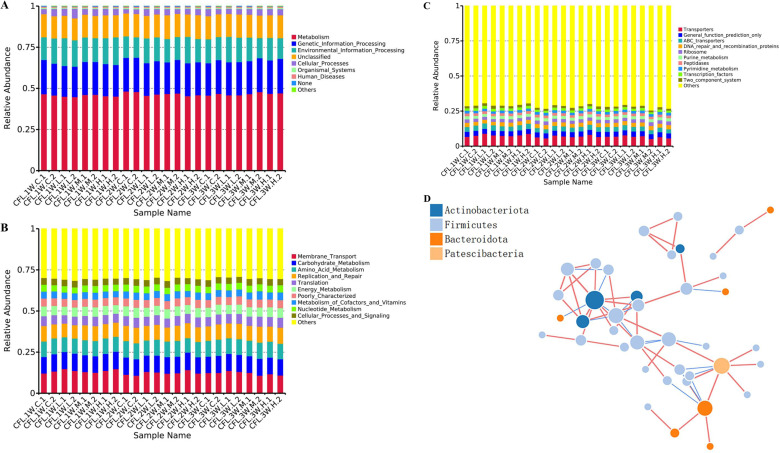
KEGG functional analysis. A. The results of functional analysis level 1 which ana ICRUST. C. The results of functional analysis level 3 which analyzed by PICRUST. D&E. The relationship of microbiota in each group after treatment of TENS. The nodes represented as genera; The node size represented as the connectivity of genera; The same color represented as the same gate level.

After 3 weeks of treatment with TENS, compared with the model control group, the top 10 functions of the changed intestinal microbiota were metabolism, genetic information processing, etc. in the level 1 (Fig 7A); The top 10 functions of the changed intestinal microbiota were membrane transport, carbohydrate metabolism, etc. in the level 2 (Fig 7B); the top 10 functions of the changed intestinal microbiota were transporters, DNA repair and recombination proteins, etc. in the level 3 (Fig 7C). After TENS treatment, the changed microbiota showed a inner relationship in a co-synergistic manner (Fig 7D, E).

## 4. Discussion

Intestinal microbiota is a complex ecosystem that keeps balance with the host and it takes participate in the regulation of inflammation, immune response and microbial metabolism [22]. This study found that microbial disorders could lead to inflammatory reactions, immune disorders and various metabolic disorders [24].

A 16S rDNA sequencing was used to be the taxonomy index for changed intestinal microbiota after TENS treatment [25]. 16S rDNA amplicon sequencing usually selects one or several variable regions, designs universal primers using conservative regions for PCR amplification, and then sequencing analysis and strain identification of hypervariable regions [26]. After sequencing, the richness and evenness of intestinal microbiota were evaluated by Alpha diversity analysis [27]. Community richness is a metric to estimate the total number of species, including the Chao 1, and observed species (the number of different operational taxonomic units, OTUs, per sample) [28]. Community diversity is a metric of relative community evenness, reflecting the richness and evenness of the species in the sample, including the Shannon index and Simpson index [28]. The decreased alpha-diversity of the gut microbiota is linked to a declined health status. This study found similar results of species diversity measures and TENS could reduce the Alpha diversity of CFL injury rats. The results of the Simpson index are different but there was no statistically significant difference in each group. Shannon index takes into account both species richness and evenness. When the community is completely uniform, the abundance of all species in the community is completely the same, the value of Shannon index reaches the maximum. Simpson index also considered species richness and evenness, but compared with Shannon index, it was more affected by evenness. It may be due to the shorter recovery period for CFL injuries and the fact that TENS did not change the Simpson Index significantly. The differences of the changed intestinal microbiota was evaluated by beta diversity and it was normally combined with dimensional reduction methods such as PCoA, NMDS, or PCA to obtain visual representations [27]. The separation rates of PC1 and PC2 were 8.27% and 6.9% respectively. There was a significant difference in the model control group and TENS groups.

After TENS treatment, compared with the the C3 (Control. 3W. CFL injury) group, the contents of *Bacteroidota, Desulfobacterota*, *Proteobacteria* and etc. of H3 (High intensity. 3W. CFL injury) group increased at the phylum; the contents of *Firmicutes*, *Patescibacteria*, *Actinobacteriota*, *Campilobacterota* and *Elusimicrobiota* of H3 (High intensity. 3W. CFL injury) group decreased. At the genus level, there were significantly different intestinal microbiota between the C3 (Control. 3W. CFL injury) group and the three TENS group. For example, the *Bacteroides*, *Lactobacillus* increased and *Ruminococcus*, *Dubosiella* decreased. These results illustrated that TENS could change intestinal microbiota.

It was reported that Urolithin A (UA) was a biologically active metabolite generated by the gut microbiota (GM), ameliorated ovariectomy-induced bone loss by suppressing osteoclast formation and the intestinal microbiota had been a targeted biomarker for diseases [29,30]. The repetitive increase in the *Ruminococcus Gnavus* is specific to spinal arthritis [31]. Studies found that the proportion of *Bacteroides Fragilis* and *Ruminococcus Gnavus* was significantly higher in athletes with a history of lateral ankle sprain (LAS), indicating a potential relationship between LAS and intestinal microbiota [21]. The increased proportion of *Ruminococcus* is consistent with our experimental results. *Ruminococcus* is a kind of gram-positive anaerobes belonging to thick-walled bacteria, which is highly correlated with inflammation, obesity, nonalcoholic fatty liver disease (NAFLD), coronary heart disease (CHD) and other diseases [32]. Compared to the model control group, the increase of beneficial *Bacteroides* occurred after treatment with TENS. As intestinal symbiotes, *Bacteroides* play a variety of roles; they protect against pathogens and provide nutrition for other microbial residents in the gut [33]. Related studies found that the ankle joint thickness of mice receiving intestinal microbiota of patients with spinal arthritis was negatively correlated with the abundance of *Bacteroides*, and *Bacteroides* is protective [34]. Studies found that the changed abundance of *Lactobacillus* is closely related to health. *Lactobacillus* is a kind of rod-shaped or spherical Gram-positive bacteria found in animals [35]. *Lactobacillus* show a bidirectional regulation in the intestinal micro-environment [36]. Strains from *Lactobacillus*, such as *Lactobacillus acidophilus* (*L. acidophilus)*, *Lactobacillus murinus* (*L. murinus)*, *Lactobacillus johnsonii* (*L. johnsonii)*, and *Lactobacillus reuteri* (*L. reuteri)*, display strong immune regulation ability [37]. *Dubosiella* is a kind of gram-positive bacteria belonging to the *Firmicutes*, is a type of probiotic bacteria, which is considered to be a kind of intestinal bacteria which can regulate metabolism, improve intestinal immunity and promote the body to resist inflammatory diseases, can affect a variety of life activities of the individual [38]. The study of intestinal microbiota in a rat model of collagen-induced arthritis showed significant enrichment of *Lactobacillus* and *Lactobacillus acidophilus* after the treatment of XiongFu powder, relieved rat hind limb joint swelling and slowed arthritis progression [37]. The diversity of intestinal microbiota is highly various because many factors, including physical, psychological, genetic, dietary, cultural and environmental determinants, affect the structure and composition of intestinal microbiota [39]. The increased beneficial bacteria was more significant than hazardous bacteria in the intestinal microbiota of CFL injury rats treated with TENS.

The intestinal microbiota of human have four phyla: *Firmicutes* (e.g., *Lactobacillus*, *Ruminococcus*)*, Bacteroidota* (*e.g., Bacteroides*), *Actinomycetes* and *Proteobacteria*, of which *Firmicutes* and *Bacteroidota* are the main components. After TENS treatment, compared with the C3 (Control. 3W. CFL injury), the phylum *Bacteroidota* increased in the M/H3 (Medium/High intensity. 3W. CFL injury) groups [40]. TENS treatment improved the intestinal microbiota composition of CFL injury rats and played a protective role in the intestinal microbiota disorder of CFL injury rats. Compared with rats in the medium-intensity and high-intensity stimulation groups, rats without TENS stimulation had a larger number of *Campilobacterota*, most of the dominant genera of untreated CFL injury rats may be *Campilobacterota*. The improvement of TENS for CFL injury could be related to cell proliferation, migration, differentiation, suppress inflammation, cartilage repair and lipid metabolism. TENS could inhibit the expression of inflammatory markers NF-κB and IL-1β, thereby alleviating the inflammatory response of CFL injury, ease pain, and improve the symptoms of CFL injury.

In this study, TENS could improve CFL injury rats and reduce IL-1β/NF-κB expressions of cartilage by regulating the IL-17 signaling pathway. The intestinal microbiota *Alloprevotella*, *Megamonas*, *Phascolarctobacteriu* was changed in CFL injury rat after TENS treatment and their function was related to anti-inflammation of IL-1β/NF-κB/IL-17 signaling pathway. This study found a new finding to investigate of intestinal microbiota for CFL injury. Studying the diversity of the intestinal microbiota could provide a potential treatments for CFL.

## 5. Conclusion

In conclusion, TENS could improve CFL injury rats and inhibit the expressions of IL-1β/NF-κB by regulating the IL-17 signaling pathway. After TENS treatment, the intestinal microbiota of CFL injury rat was changed including the increase of *Bacteroidetes, Desulfobacterota, Proteobacteria, Cyanobacteria, Firmicutes and Patescibacteria*, etc. and the decrease of *Alloprevotella*, *Megamonas*, *Phascolarctobacteriu*. And the changed intestinal microbiota were related to CFL injury.

## Supporting Information

Supporting information.zip (ZIP)

## Abbreviations

ACM: Alternative and complementary medicine
BMD: Bone mineral density
BV/TV: Bone volume fraction
CFL: Calcaneofibular ligament
GM: Gut microbiota
IL-1β: Interleukin-1β
IL-17: Interleukin-17
KEGG: Kyoto encyclopedia of genes and genomes
LAS: Lateral ankle sprain
Micro-CT: Micro-computed tomography
NF-κB: Nuclear factor kappaB
NMDS: Non-metric multi-dimensional scaling
NSAIDs: Non-steroidal anti-inflammatory drugs
OTU: Operational taxonomic unit
PCoA: Principal coordinate analysis
TENS: Transcutaneous electrical nerve stimulation
Tb.n: Trabecular number
Tb.th: Trabecular thickness
UPGMA: Unweighted pair-group method with arithmetic mean
