# Supplementary material for: TENS improves CFL injury rat and regulates the intestinal microbiota
Source: PLoS One. 2025 Apr 3;20(4):e0319592. doi: 10.1371/journal.pone.0319592 (PMC11967936; doi:10.1371/journal.pone.0319592)
Supplement: Supporting information.zip — (ZIP) [file pone.0319592.s001.zip › Full title page.docx]

**TENS improved CFL injury by regulating the intestinal**

**Intestinal microbiota could becomes a potential CFL treatment**

Min Miao ^a,b^, Tong Ma ^a,b^, Ran Chen ^c^, Kuan Geng ^d^, Zhiqiang Shen ^a,b*^, Yan Sun ^a,b*^

(a. Pharmaceutical College & Key Laboratory of Pharmacology for Natural Products of Yunnan Province, Kunming Medical University, Kunming Yunnan, 650500, P.R. China)

(b. The College of Modern Biomedical Industry, Kunming Medical University, Kunming, 650000, Kunming Yunnan, P.R. China)

(c. Clinical Lab, The Second Affiliated Hospital of Kunming Medical University, Kunming Yunnan, 650000, P.R. China)

(d. Department of Radiology, The First People's Hospital of Honghe State, Honghe, 661600, Mengzi Yunnan, P.R. China)

Min M., **contact e-mail:** 1228594054@qq.com; Tel: +86-15187861912.

Tong M., **contact e-mail:**2893258854@qq.com; Tel: +86-18313591410.

Ran C., **contact e-mail:** 993403050@qq.com; Tel: +86-13888119406.

Geng K., **contact e-mail:** gengkuan1018@163.com.

***Corresponding author:**

Dr. Zhiqiang Shen, Pharmaceutical College & Key Laboratory of Pharmacology for Natural Products of Yunnan Province, Kunming Medical University, Kunming Yunnan, 650500, China. Tel:86-13888400622, E-mail: shzhq21cn@qq.com ;

Dr. Yan Sun, Pharmaceutical College & Key Laboratory of Pharmacology for Natural Products of Yunnan Province, Kunming Medical University, Kunming Yunnan, 650500, China. Tel: 86-18487369694, E-mail: 553046530@qq.com.

**Acknowledgements**

Not applicable.

**Funding**

This study was supported by the Applied Basic Research Foundation of Yunnan Province 202101AU070258/2022J0645, the Joint Foundation of Department of Science and Technology of Yunnan Province 202201AY070001-008, the Yunnan Provincial Department of Education Science Research Fund Project 2023J0256, the Research project on undergraduate educational and teaching reforms in Yunnan province JG2023001. The above institutions did not participate in the design of the study, collection, analysis, interpretation of data, or in writing the manuscript.

**Data Availability**

The datasets generated and analysed during the current study are available in the [NCBI] repository, [BioProject accession number：PRJNA1176105], the rest of the data has been uploaded to [Mendeley Data], access link for https://data.mendeley.com/preview/7k6dwybgg4?a=015d20fe-c04e-433a-bead-d641f3578d37, Reserved for the DOI: 10.17632/7k6dwybgg4.1

**Consent for publication**

Not applicable

**Conflict of interests**

To the best of our knowledge, the named authors have no conflict of interest, financial or otherwise, including MM, TM, RC, KG, YS, ZQS.

**Authors' contributions**

ZQS and YS designed this study. MM, TM, RC, KG finished these experiments. MM analyzed data. MM drafted this manuscript. ZQS and YS revised this manuscript.
