# Supplementary material for: TENS improves CFL injury rat and regulates the intestinal microbiota
Source: PLoS One. 2025 Apr 3;20(4):e0319592. doi: 10.1371/journal.pone.0319592 (PMC11967936; doi:10.1371/journal.pone.0319592)
Supplement: Supporting information.zip — (ZIP) [file pone.0319592.s001.zip › animal resource.pdf]

昆明医科大学医学伦理委员会
审查批件

批件号：KMMU2023MEC203

项目名称	经皮神经电刺激治疗踝关节运动损伤的研究		
申办者单位	昆明医科大学	负责人	孙岩
合作单位			
审查类别	初始审查		
审查文件	1. 伦理审查申请表；<br>2. 研究方案；<br>3. 向受试者提供的书面研究简介；<br>4. 知情同意书；<br>5. 主要研究者专业履历。		
审查意见	依据《涉及人的生物医学研究伦理审查办法》（中华人民共和国国家卫生和计划生育委员会令第 11 号）、《赫尔辛基宣言》和《昆明医科大学医学伦理委员会章程》的伦理原则与要求，经本伦理委员会审查，意见如下：<br>1. 主要研究者符合要求。<br>2. 知情同意书内容符合相关要求。<br>3. 研究方案基本考虑了伦理原则，**同意修改后执行**。<br>4. 建议研究方案修改：<br>①完善知情同意：完全告知风险（参与者采血时可能引起的晕血等不适症状、参与者进行电刺激时引起的局部皮肤反应）<br>②		
审查委员	刘佳 晏姗 张瑞宏 李彩霞 王玉明		
伦理委员会主任委员签字：[signature] 日期：2024.1.9<br>昆明医科大学医学伦理委员会（盖章）			
备注	1. 修改后同意/重审项目，应将修改后文件及时反馈伦理委员会，进行进一步审查。<br>2. 不同意/终止或暂停项目，批件发出 2 周内可向伦理委员会就有关事项做出解释或提出申诉。<br>3. 实验研究应严格按照本伦理委员会批准的文件执行，在实验实施过程中，如实验方案、知情同意书等文件有任何修改，应及时向本伦理委员会提交变更申请，补充更新文件，经伦理委员会重新审查批准后，方可执行。<br>4. 如发生严重不良事件、可能影响风险受益的任何事件或新信息应及时报告伦理委员会。		
