# Supplementary figures and images for: TENS improves CFL injury rat and regulates the intestinal microbiota
Source: PLoS One. 2025 Apr 3;20(4):e0319592. doi: 10.1371/journal.pone.0319592 (PMC11967936; doi:10.1371/journal.pone.0319592)

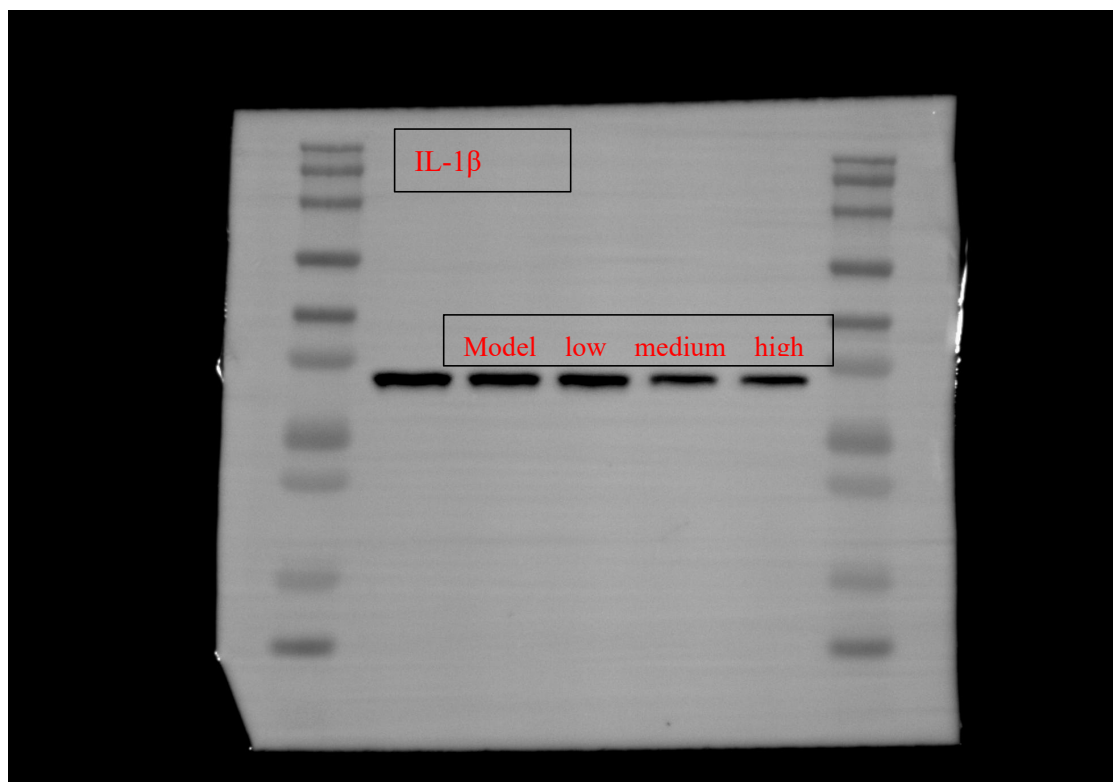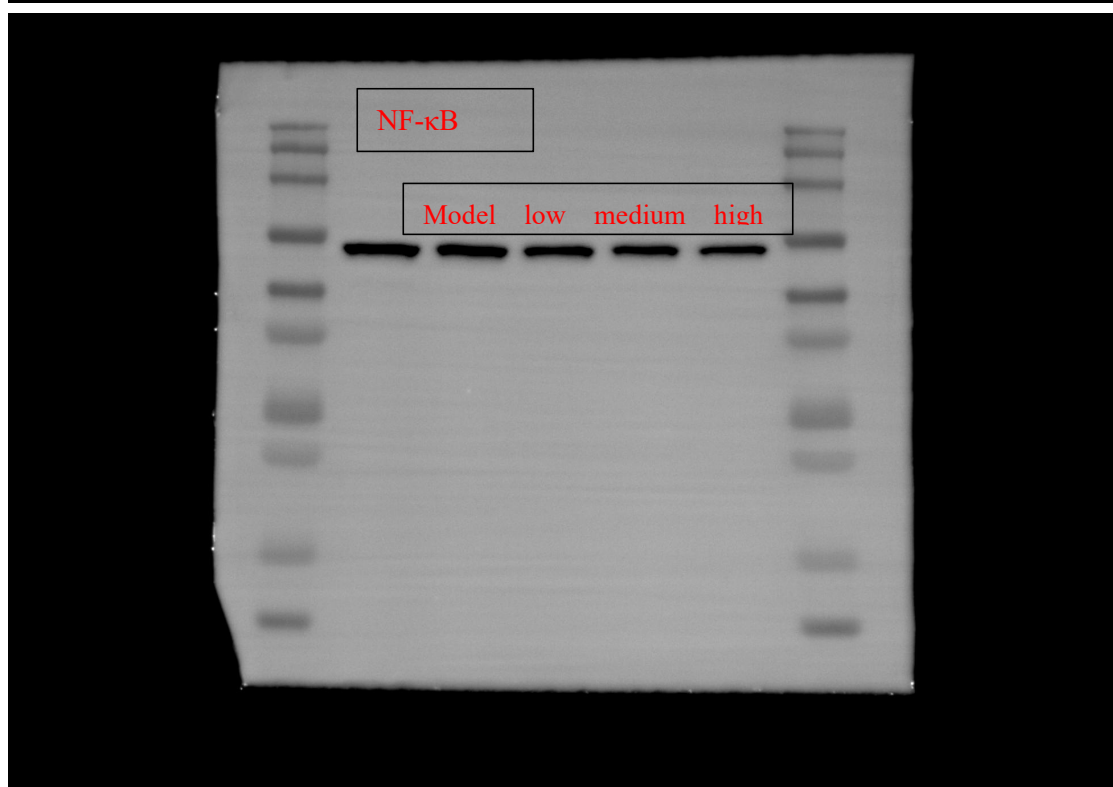

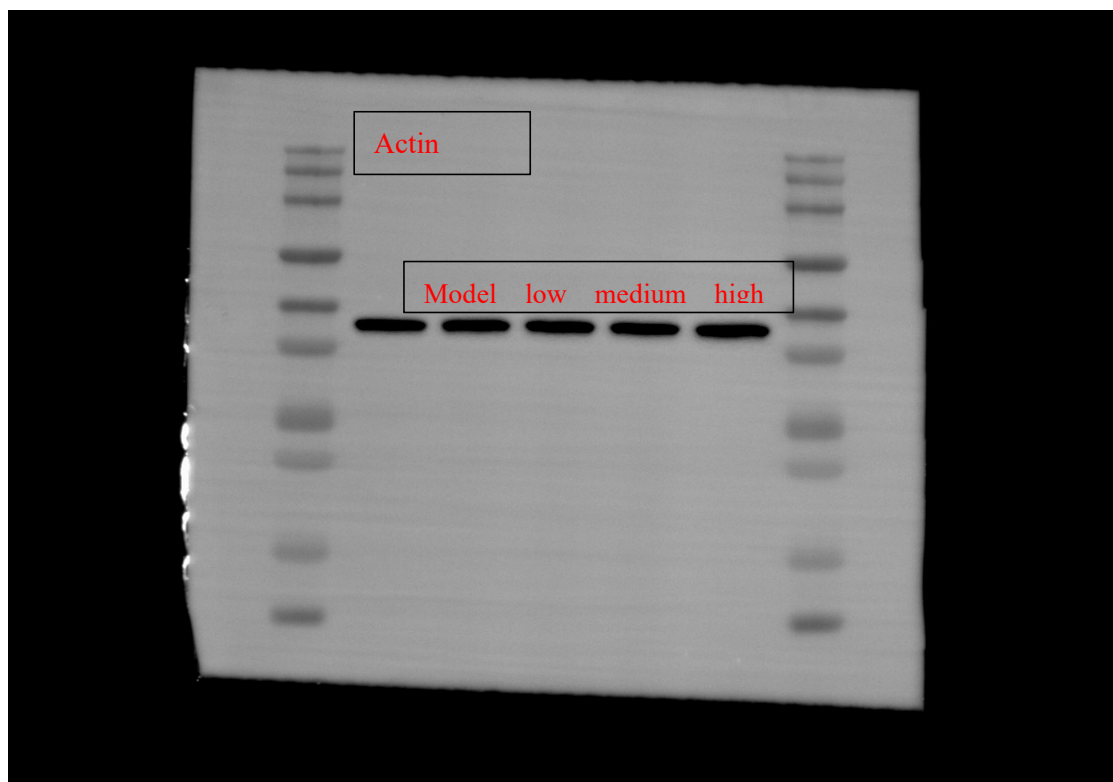

Supplement: Supporting information.zip — (ZIP) [file pone.0319592.s001.zip › blots.pdf]
